# Predicting cytotoxicity from heterogeneous data sources with Bayesian learning

**DOI:** 10.1186/1758-2946-2-11

**Published:** 2010-12-09

**Authors:** Sarah R Langdon, Joanna Mulgrew, Gaia V Paolini, Willem P van Hoorn

**Affiliations:** 1 Department of Chemistry and Biology, Pfizer Global Research and Development, Sandwich Laboratories, Sandwich, Kent, CT13 9NJ, UK; 2 Department of Biology, Pfizer Global Research and Development, Sandwich Laboratories, Sandwich, Kent, CT13 9NJ, UK; 3 In Silico Medicinal Chemistry, Cancer Research UK Cancer Therapeutics Unit, The Institute of Cancer Research, Haddow Laboratories, 15 Cotswold Road, Sutton, Surrey, SM2 5NG, UK; 4 Gaia Paolini Ltd, 29 High Street, Bridge, Canterbury, CT4 5JZ, UK; 5 Accelrys Ltd, 334 Cambridge Science Park, Cambridge, CB4 0WN, UK

## Abstract

**Background:**

We collected data from over 80 different cytotoxicity assays from Pfizer in-house work as well as from public sources and investigated the feasibility of using these datasets, which come from a variety of assay formats (having for instance different measured endpoints, incubation times and cell types) to derive a general cytotoxicity model. Our main aim was to derive a computational model based on this data that can highlight potentially cytotoxic series early in the drug discovery process.

**Results:**

We developed Bayesian models for each assay using Scitegic FCFP_6 fingerprints together with the default physical property descriptors. Pairs of assays that are mutually predictive were identified by calculating the ROC score of the model derived from one predicting the experimental outcome of the other, and vice versa. The prediction pairs were visualised in a network where nodes are assays and edges are drawn for ROC scores >0.60 in both directions. We observed that, if assay pairs (A, B) and (B, C) were mutually predictive, this was often not the case for the pair (A, C). The results from 48 assays connected to each other were merged in one training set of 145590 compounds and a general cytotoxicity model was derived. The model has been cross-validated as well as being validated with a set of 89 FDA approved drug compounds.

**Conclusions:**

We have generated a predictive model for general cytotoxicity which could speed up the drug discovery process in multiple ways. Firstly, this analysis has shown that the outcomes of different assay formats can be mutually predictive, thus removing the need to submit a potentially toxic compound to multiple assays. Furthermore, this analysis enables selection of (a) the easiest-to-run assay as corporate standard, or (b) the most descriptive panel of assays by including assays whose outcomes are not mutually predictive. The model is no replacement for a cytotoxicity assay but opens the opportunity to be more selective about which compounds are to be submitted to it. On a more mundane level, having data from more than 80 assays in one dataset answers, for the first time, the question - "what are the known cytotoxic compounds from the Pfizer compound collection?" Finally, having a predictive cytotoxicity model will assist the design of new compounds with a desired cytotoxicity profile, since comparison of the model output with data from an *in vitro *safety/toxicology assay suggests one is predictive of the other.

## Background

A 2003 study estimated the cost of the research and development of a drug up to the pre-approval point to be over 800 million US dollars [1]. Toxicity is the reason behind the withdrawal of over 90% of drugs from the market and the failure of a third of drugs in phase I-III clinical trials [2]. Because of the huge cost in researching and developing a new drug, pharmaceutical companies want to minimise the number of failures in clinical trials and the number of withdrawals from the market. One way to minimise the number of failures is to ensure drugs are not toxic before they reach clinical trials. This is done by screening compounds for toxicity in the early stages of drug discovery and understanding the mechanisms of toxicity to avoid designing toxic drugs in the first place.

The general toxicity testing pipeline in the pharmaceutical industry begins with *in vitro *toxicology screening followed by *in vivo *studies [3]. The majority of mandatory non-clinical toxicity investigations are *in vivo *[4]. Preclinical *in vivo *studies are used to determine potential adverse effects of drugs, estimate safety margins [5], understand mechanisms of toxicity and decide if compounds should be eliminated from the development process [6]. At the moment no *in vitro *test for acute oral toxicity has been approved by regulatory agencies to be sufficient evidence to allow commencement of clinical trials [4]. However, there are two mandatory *in vitro *studies, genotoxicity and hERG assays, that must be carried out before clinical trials can commence.

In order to use *in vivo *and *in vitro *methods, compounds must have already been synthesised and available in sufficient quantities. Moreover, the experimental methods are time consuming and costly. For the time being it is a requirement that *in vitro *and *in vivo *toxicity studies are carried out on all drug candidates before they reach clinical trials. Development of a predictive model allows *in-silico *screening of compounds in virtual libraries, *i.e. *before any compounds are actually made.

*In vitro *cytotoxicity assays are often run in parallel to primary cell-based activity screens in order to identify hits that only appear to be active because of their cytotoxic effects [7,8]. These cytotoxicity assays are usually run to triage compounds which appear active in a cell-based primary assay against a target of interest. The choice of cytotoxicity assay is not restrictive, with some scientists choosing to re-use an assay from a previous project, while others opt for the newest cytotoxicity assay kits on the market. Cytotoxicity assays may be run against cell lines from different species (*e.g. *human, mouse, rat) and/or different cell types (*e.g. *skin, neuronal, liver). The choice of cell line and/or species may be aligned to those used in the primary target assay or be more comparable to the *in vitro *toxicology assay which it precedes. Assay methodologies vary widely (*e.g*. measurements of mitochondrial activity, ATP concentrations, and membrane integrity) but the basic principle is to assess cell viability and/or proliferation. Endpoint detection methods are similarly diverse, *e.g. *luminescence, absorbance or fluorescence. Finally, the period of cell incubation with compound varies from 2 hours in acute studies to several days in some long term antiviral assays. Again the length of incubation time may be selected simply to parallel that of the primary assay.

The aim of this project was to develop a computational model which could be used to generate a general "cytotoxicity score". This could then be used as a service to alert when a new synthesis is similar to a known cytotoxic compound, and/or as a tool to give an indication of compound cytotoxicity. To make this model as generally applicable as possible we tried to maximise the coverage of chemical space in the training set by merging data from multiple assays. We see a general cytotoxicity model as crucial in early stages of drug discovery when typically chemical series are pursued for which little cytotoxicity data is available and therefore no opportunity exists to build a more accurate series-specific model. Users could then access more information to include cell line, species, compound dose and incubation time details - and use this to triage their data further. Finally, we plan to collaborate with safety colleagues to be able to identify the cytotoxicity assays which are the best predictors of *in vitro *and clinical toxicity. This would provide the potential to reduce compound attrition since series with cytotoxic characteristics which track with known toxicology profiles would not be pursued.

Predicting toxicity is a challenging task because of the complex biological mechanisms behind it. The results of *in vivo *studies can be used to validate *in vitro *studies [9]. As long as the *in vitro *methods used to generate the data are successful at predicting *in vivo *outcomes, then the *in silico *models built with that data should be able to closely mimic the results of *in vivo *studies [9]. In this project, data from *in vitro *experiments will be used alongside Bayesian learning to predict the cytotoxicity of compounds.

There are several examples of predicting cytotoxicity from *in vitro *data in the literature, including the use of neural networks [10], random forests [11], decision trees and linear least squares [12]. The last example successfully predicts general cytotoxicity using *in vitro *results from 59 different cell lines. In this work we will attempt to predict general cytotoxicity using *in vitro *data gathered using many different assay formats, we will also compare our work with Guha and Schürer's random forests, as we can reproduce their models using our own methods and the same publicly available datasets.

Bayesian learning is a popular and mature machine learning method that can be used to classify molecules in two sets *e.g. *active/inactive or toxic/non-toxic. It has many applications in the pharmaceutical industry including modelling biological activity [13-15], such as kinase inhibitors [16] and hERG blockers [17,18], enriching high throughput screening (HTS) data [19,20] & docking results [21], predicting combinatorial library protocols [22] and describing compound similarity [23]. Bayesian learning is used in this paper because of its speed, safety with respect to over-fitting and its ability for handling noisy data. The speed of Bayesian learning scales linearly with the number of compounds, making it a fast and efficient technique. No pre-selection of descriptors is required prior to learning as only those descriptors that correlate with activity will have a great effect on learning and unimportant descriptors will not lead to over-fitting. This also means that Bayesian learning performs well with noisy data, as is the case in this study which has a large amount of primary assay data and an expected high number of false positives and negatives.

Another advantage of Bayesian learning is that it does not require the active/inactive ratio in the training set to be balanced; instead, the assumption is that the ratio present in the training set is representative of the ratio in the set where predictions are to be made. Therefore pre-processing to derive a training set with balanced active/inactive data is not required.

We have used Bayesian learning with publicly available and in-house cytotoxicity assay data to predict the cytotoxicity of compounds.

We start by discussing the use of Bayesian learning to model cytotoxicity using publicly available data and the validation of these methods. Next we describe the application of these methods to a much larger Pfizer in-house data set collected from multiple different assays. Prediction networks, based on the ability of assay data to predict the results of other assays are generated and then used to select assay data suitable as a training set for a general cytotoxicity model.

## Results and Discussion

### Modelling Public Data

Two publicly available cytotoxicity datasets were downloaded from PubChem [24]: "Scripps" which contained a mixture of single point (percent inhibition) primary data and IC50 confirmation data and "NCGC" which contained only IC50 data [25]. These datasets have previously been used by Guha and Schürer to derive Random Forest models [11]. For each data set, two versions of Bayesian models have been built using different descriptors. The FCFP_6 models used FCFP_6 fingerprints, AlogP, number of hydrogen bond donors, number of hydrogen bond acceptors, number of rotational bonds and molecular fractional polar surface area as descriptors. The BCI models used BCI-1052 structural keys as descriptors, as used in the published Random Forest models [11]. We were not able to calculate the BCI fingerprints for all compounds therefore some compounds were left out (11 from the NCGC data, 33 from the Scripps IC50 data and 3800 from the Scripps percent inhibition data). For each model, the data set was split into 5 equal-sized random sets. The models were built on 4 of these sets (80% of the data) and tested with the remaining 20%. This process was repeated so that 5 models were built, each tested on the set that was left out of the training data. This is a technique known as 5-fold cross-validation. For each validation a receiver operating characteristic plot (ROC plot) and truth table were generated. The models' performance can be assessed from the average ROC plot and truth table for the 5 models.

### Scripps IC50 Data

**Figure 1:**
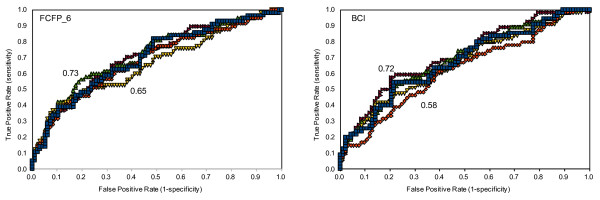
**ROC scores for the 5-fold cross validation of the Scripps IC50 FCFP_6 (left) and BCI (right) models**. Minimum and maximum ROC scores are shown.

We built a Bayesian model with a potency cut-off of 5.5, in accordance with Guha and Schürer [11]. This means that all molecules with pIC50 > 5.5 were considered cytotoxic. A ROC plot charts the false positive rate of a model versus its true positive rate and represents the cost-benefit trade-off [26]. The area under the curve is the ROC score: the probability that the model will correctly identify an active molecule from a randomly selected pair consisting of an active and an inactive molecule. A perfect model will have a ROC score of 1 corresponding to 100% true positive (TP) rate, and 0% false positive (FP) rate, while a random model will have a ROC score of 0.5 as there is a 50% chance of correctly classifying the active molecule from the pair. In Figure 1 the ROC scores for the 5 fold validation of the Scripps FCFP and BCI models are shown. The average ROC scores and standard deviations in the ROC score for the FCFP_6 and BCI models are 0.70 ± 0.03 and 0.66 ± 0.05, respectively. These scores indicate the models are poorly predictive and show no clear advantage or disadvantage for using either descriptor. The truth tables and derived specificity/selectivity data in Tables 1 and 2 show that although the specificity is fairly high, the sensitivity is rather poor, suggesting that the cut off for cytotoxicity is too low. The published Random Forest model by Guha and Schürer performed comparably to the Bayesian model described here [11]. Their sensitivity of 0.56 is comparable to our BCI model at 0.53 ± 0.10, but the Random Forest model is better at predicting inactivity with a specificity of 0.80 compared to the value of 0.68 ± 0.03 we obtained. The Random Forest model has a ROC score of 0.73. This is higher than the average score achieved here (0.66 ± 0.05), although for one of our five sets a close score of 0.72 was obtained, this illustrates the need for multiple cross-validation. The results here show that the performance of a model depends on the training set, as different ROC scores were obtained for each of the 5 validations. If the Random Forest model had been cross-validated multiple times an average closer to ours may have been obtained.

**Table 1:** Truth table for 5-fold cross-validation of the Scripps IC50 FCFP_6 and BCI models

	Scripps IC50 FCFP_6 Model		Scripps IC50 BCI Model	
Experiment	Cytotoxic	Non-toxic	Cytotoxic	Non-toxic
Cytotoxic	21 ± 1.5%	16 ± 1.6%	20 ± 3.7%	17 ± 3.7%
Non-toxic	18 ± 0.9%	45 ± 0.9%	20 ± 2.1%	43 ± 2.1%

**Table 2:** Specificity and sensitivity of Scripps IC50 FCFP_6 and BCI models

	Scripps IC50 FCFP_6 Model		Scripps IC50 BCI Model	
	Cytotoxic (sensitivity)	Non-toxic (specificity)	Cytotoxic (sensitivity)	Non-toxic (specificity)
Fraction correctly classified	0.57 ± 0.04	0.71 ± 0.01	0.53 ± 0.1	0.68 ± 0.03

### Scripps Percent Inhibition Data

**Figure 2:**
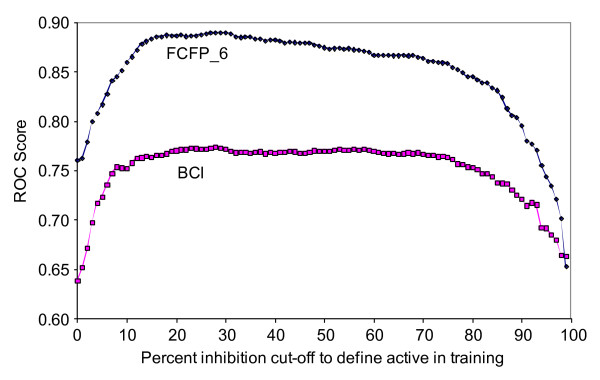
**ROC score and percent inhibition cut-off for toxicity while training a Bayesian model**. Each point represents a different model. The ROC scores are calculated using all compounds from the percent inhibition data set with actives defined as pIC50 > 5.5

In an attempt to increase the performance of their model, Guha and Schürer added 10,000 molecules classed as non-toxic from the Scripps percent inhibition data set to the IC50 training set [11]. The reasoning behind this was to emphasise the difference between the two classes. We used a similar approach to improve our model, but included the entire Scripps percent inhibition data set in the training set (59,780 measurements). The Scripps percent inhibition data came from two assays (PubChem AID 364 and 463). The cut-off value above which a compound was considered toxic was calculated by taking the the average percent inhibition of all compounds tested plus three times the standard deviation. This equates to 39% in assay 364 and 30% in assay 463. However, not all of these compounds were available for submission to the corresponding IC50 assay and therefore some less active compounds were submitted instead. Applying a cut-off to classify a compound as toxic or non-toxic is arbitrary. There is no expectation that the toxicity differs significantly between a compound with a percent inhibition just above the cut-off and one with a cut-off just below, especially when taking into account the experimental error. The optimal cut-off can be determined by the desire to see as few false positives as possible in the IC50 confirmation assay, for which there typically exists a resource constraint limiting the number of compounds that can be submitted. Choosing a high percent inhibition cut-off like mean plus three standard deviations will limit the number of hits and the false positives amongst them, thereby ensuring a large proportion of compounds will pass the confirmation assay. However, the cost of building a Bayesian model is independent on the cut-off, in fact the cost of model building is low enough that the optimal cut-off can be found by building multiple models and choosing the best model according to a predefined metric. This idea was suggested by David Rogers [27]. This method assumes that the actives found in the IC50 confirmation assays are the true actives that can be found in the entire data set. All 100 models with percent inhibition cut-offs ranging from 1 to 100 were built. For each model, the ROC score was calculated for predicting the toxic compounds found in the IC50 assay as positives (toxic) and all other compounds from the HTS as negatives (non-toxic). In Figure 2 the ROC score is plotted against the percent inhibition cut-off. The optimum cut-off is 29% (ROC 0.89) for the FCFP_6 model and 28% (ROC 0.77) for the BCI model. This is close to the cut-off of 30% that was applied in assay 463, which is not surprising since this assay contributed ~17 times as many measurements as assay 364. However, the curves in Figure 2 are nearly flat; a similarly predictive model can be obtained using any cut-off between ~10% and ~80%. This was also observed previously by Rogers, who speculated that this could be used to exploit structure-activity relationships (SAR) that exist mostly or entirely in the region of low (below the cut-off) percent inhibition. Bayesian modelling is biased towards compound sets displaying clear SAR, *i.e. *actives that are part of a series of chemically similar compounds. By lowering the cut-off many random false positives will be included but, as long as enough additional members of the various SAR series are added, the model will improve or at least not deteriorate. The mean percent inhibition of all 59780 measurements was 1.2% with a standard deviation of 9.8%. The lower viable cut-off for toxicity (10%) is therefore close to just one standard deviation from the mean. The FCFP_6 models clearly outperform the BCI models, which is not unexpected since the FCFP_6 fingerprints contain far more features than the BCI fingerprints and the FCFP_6 models were built using additional physical property descriptors.

In Tables 3 and 4 the results are presented when the Scripps percent inhibition models derived with optimised cut-offs are applied to the IC50 data set. Models were derived from percent inhibition data and evaluated using IC50 data as in Table 1.

**Table 3:** Truth table for Scripps percent inhibition FCFP_6 and BCI models.

	Scripps percent inhibition FCFP_6 model		Scripps percent inhibition BCI model	
Experiment	Cytotoxic	Non-toxic	Cytotoxic	Non-toxic
Cytotoxic	30 ± 3.4%	7 ± 1.7%	27 ± 2.6%	11 ± 0.7%
Non-toxic	41 ± 2.0%	22 ± 3.3%	40 ± 3.2%	23 ± 3.1%

**Table 4:** Specificity and sensitivity of Scripps percent inhibition FCFP_6 and BCI models

	Scripps percent inhibition FCFP_6 model		Scripps percent inhibition BCI model	
	Cytotoxic (sensitivity)	Non-toxic (specificity)	Cytotoxic (sensitivity)	Non-toxic (specificity)
Fraction correctly classified	0.82 ± 0.05	0.35 ± 0.04	0.72 ± 0.03	0.37 ± 0.05

The prediction accuracy of cytotoxic compounds expressed by the sensitivity has increased markedly compared to models derived previously from IC50 data: but this was achieved at the cost of a decreased specificity. In contrast, Guha and Schürer did not obtain an appreciable difference in the sensitivity (or specificity) when adding 10,000 non-toxic compounds to the training set [11]. The cost of the increased sensitivity in our model is a much higher rate of false positives. However, of the 484 compounds classified as non-toxic (pIC50 ≤ 5.5), nearly half (231) could be classified as moderately toxic since they possess an IC50 ≤ 10 μM (pIC50 ≥ 5). The majority of these (151) are predicted as toxic by the FCFP_6 model (model score >0). For the BCI model similar numbers were obtained (463 compounds classified as non-toxic compounds, 223 moderately toxic, 136 with Bayesian score >0). Both Bayesian models derived from the Scripps percent inhibition data are good at picking compound series with toxicity issues but not as good at differentiating which member of the series is toxic and which one is not. This is illustrated in Table 5 where a series of 4 compounds is shown all of which are predicted toxic. Only one of these (CID 659940) actually has a pIC50 value above 5.5 but the toxic prediction counts it as true active. The other three are counted as false positive. However, it should also be noted that Guha and Schürer lowered the pIC50 cut-off for toxicity to 4.68 when they compared Scripps data with NCGC data [11]. Our main aim is to derive a model that highlights *potentially *problematic series early on in the drug discovery process and in this context *one *false positive such as compound 663916 which is a very close analogue to the moderately cytotoxic compound 664633 would not necessarily indicate failure of the model. Indeed, a slightly higher false positive rate could be considered an advantage when using the model output as a compound triage tool for deleterious safety effects.

**Table 5:** Example series of compounds which are all predicted to be toxic (Scripps percent inhibition FCFP_6 model score >0).

CID	663916	664633	664724	659940
				

pIC50	<4.40 (non-toxic)	5.24 (moderate toxic)	4.84 (non-toxic)	5.64 (toxic)

Score	36.80	28.77	41.14	42.45

Only one is truly toxic as defined by Guha and Schürer [11], but two still have a measurable pIC50. All of these were hits in the percent inhibition assay (min percent inhibition was 53% obtained for 664724).

### NCGC Data

**Figure 3:**
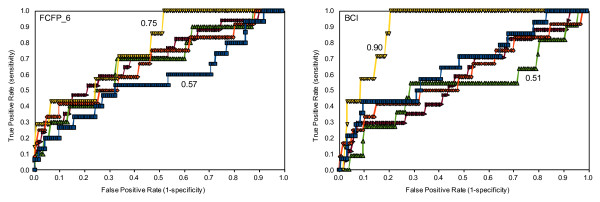
ROC plots of 5-fold cross-validated NCGC IC50 FCFP_6 (left) and BCI (right) models

As with the Scripps IC50 data, 5-fold cross-validated FCFP_6 and BCI Bayesian models were built from the NCGC Jurkat IC50 data set. The cut-off for cytotoxicity was set at pIC50 > 4.64 to enable comparison of our results to Guha and Schürer [11]. In Figure 3 the ROC plots for the 5-fold cross-validation are shown. The average ROC scores and standard deviations in the ROC score for the FCFP_6 and BCI models are 0.67 ± 0.07 and 0.65 ± 0.15, respectively, which indicates poor model performance, similar to the results obtained for the Scripps data. As was the case with the Scripps IC50 data, there is no appreciable difference between the BCI and the FCFP_6 models apart from the much larger variation of the ROC score for the BCI model. This can be explained by the lower number of toxic molecules in this dataset: with 5-fold cross validation there are on average 12 toxic compounds present in each test set. Tables 6 and 7 illustrate that the classification of cytotoxic molecules as expressed by the sensitivity is again low, but the specificity is high.

**Table 6:** Truth table for the 5-fold cross-validation of the NCGC IC50 FCFP_6 and BCI models

	NCGC IC50 FCFP_6 model		NCGC IC50 BCI model	
Experiment	Cytotoxic	Non-toxic	Cytotoxic	Non-toxic
Cytotoxic	1.5 ± 0.3	3.3 ± 1.2	1.7 ± 0.6	2.9 ± 1.4
Non-toxic	8.7 ± 2.0	86.6 ± 2.8	13.5 ± 1.8	81.9 ± 2.3

**Table 7:** Specificity and sensitivity of the NCGC IC50 FCFP_6 and BCI models.

	NCGC IC50 FCFP_6 Model		NCGC IC50 BCI Model	
	Cytotoxic	Non-toxic	Cytotoxic	Non-toxic
Fraction correctly classified	0.32 ± 0.06	0.91 ± 0.02	0.41 ± 0.20	0.86 ± 0.02

**Figure 4:**
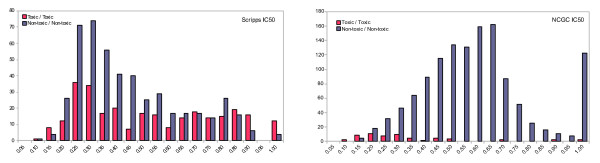
**Histograms of FCFP_6 Tanimoto internal similarity distribution**. Toxic (red) and non-toxic (blue) compounds are shown for the Scripps IC50 set (left) and NCGC IC50 set (right). For each compound, the highest similarity score was kept to any other compound in the same (toxic/non-toxic) class. In the Scripps set the toxic compounds are on average more similar to each other than the non-toxic compounds. In the NCGC set the opposite is the case, toxic compounds do resemble each other less than non-toxic compounds. Similar distributions were obtained with BCI fingerprints. A similarity of 1 does not necessarily imply compounds are identical

The low sensitivities of the Scripps and NCGC IC50 models are not a result of the percentage of toxic compounds in the data set since the Scripps IC50 set contained 37% toxic compounds while the hit rate of the NCGC set was much lower at 4.6%. Furthermore Guha and Schürer have selected compounds in their training sets to have a toxic/non-toxic ratio of 1/1 [11], yet they also obtained models with low sensitivity. A compound which is cytotoxic can be so via multiple mechanisms - a fundamental difference when comparing with single endpoint toxic mechanisms like hERG or P450 inhibition [17]. While for the latter category a single pharmacophore can be derived, this is not possible for cytotoxicity as a model of cytotoxicity is in effect a collection of models for each of the different toxicity mechanisms that result in the measured endpoint. To illustrate this point, even though the biological assay data for the Scripps and NCGC compounds was actually obtained from experiments using the same cell line (Jurkat) and measured cell viability determined by ATP concentration, the sensitivity of the NCGC model (0.32) is much lower than the sensitivity of the Scripps IC50 model (0.57). The most likely explanation is that the two compound sets act via different mechanisms to achieve the same endpoint - a reduction in ATP levels. This hypothesis is strengthened further upon examination of the different similarity distributions of both sets of compounds. In Figure 4 the internal similarity of toxic compounds is compared to the internal similarity of non-toxic compounds. For each compound, the FCFP_6 Tanimoto similarity scores were calculated versus all other compounds in the same class (toxic or non-toxic) and the highest value was retained. The toxic compounds in the Scripps IC50 set are more similar to each other (average similarity 0.52) than the non-toxic compounds (average similarity 0.44), while the opposite is the case for the NCGC set (average similarity 0.33 toxic versus 0.59 non-toxic). The toxic compounds in the NCGC set are less like each other than in the Scripps set which makes prediction of toxicity more difficult for the NCGC set.

### Cross Predictions Between Scripps And NCGC

**Figure 5:**
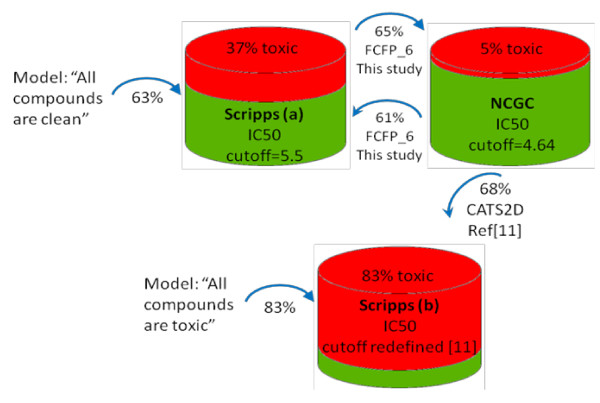
**Illustration of cross-predictive power for a number of models derived from public datasets**. Trivial models ("All compounds are clean" and "All compounds are toxic") are compared to models developed in this study ("This study") and in reference [11] ("Ref[11]"). Arrows indicate the direction of prediction. The percentage shown below each arrow is the percentage of correctly classified compounds: (true positives+ true negatives)/all. The toxicity cutoff of the Scripps dataset (b) was defined in ref[11] resulting in 83% toxic compounds. Toxic and non-toxic sets are shown in red and green, respectively.

In Figure 5 we show the results of using models derived from the public datasets to cross-predict each other, compared with predictions from Ref. [23] and from the trivial "all toxic" and "all non-toxic" models.

Firstly, we tested the NCGC Jurkat IC50 models (FCFP_6, toxicity cut-off pIC50 > 4.64) against the Scripps IC50 dataset (FCFP_6, toxicity cut-off pIC50 > 5.5). The NCGC models don't distinguish toxic from non-toxic, as indicated by the quasi-random ROC scores at 0.52 (the BCI model was no better at 0.50). In ref [23], Guha and Schürer considered their NCGC model predictive, but only after altering the fingerprint descriptors (CATS2D) used to train the model, and applying a different toxicity cut-off to the Scripps set, resulting in 640 out of 775 compounds being toxic (about 83%). They did not report a ROC score, but a percentage of correctly classified compounds (68%). This is shown in Figure 5 together with the value of 61% we obtained against the Scripps set with the original cut-off of 5.5. The model in ref [23] had a high sensitivity (0.76) and a low specificity (0.26); in effect the model was successful by predicting most compounds to be toxic - possibly as a consequence of forcing down the cut-off. The model "all compounds are toxic" would have correctly classified 83% of the compounds. Our FCFP_6 model can be considered the reverse. With the original cut-off for toxicity (pIC50 > 5.5) the sensitivity is low (0.08) and the specificity is high (0.92); this model yielded a 61% correct classification by predicting the majority of compounds to be non-toxic. The simplistic "all compounds are non-toxic" model would have correctly classified 63% of the compounds. As illustrates, the two trivial models would perform better than the models reported by Guha and Schürer and ourselves, indicating that our models failed at predicting each other. We also tried to predict the NCGC outcomes by models from the Scripps dataset. Again, the models derived from the Scripps IC50 could not correctly classify the NCGC set, as shown by the ROC scores of 0.51 (FCFP_6) and 0.40 (BCI). The ROC scores improved to 0.60 (FCFP_6) and 0.51 (BCI) when the Scripps percent inhibition models were used, but not enough to indicate good predictive power. Figure 5 shows the percentage of correct prediction (65%) of the FCFP_6 model.

**Figure 6:**
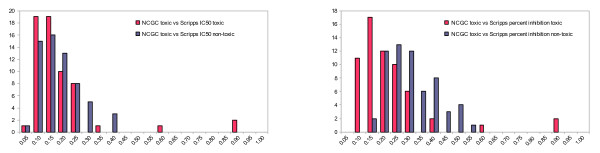
**FCFP_6 Tanimoto similarity between the toxic compounds from NCGC and the Scripps compounds**. In the Scripps data set, toxic compounds are red, non-toxic compounds, blue. In the histogram on the left the Scripps IC50 set was used as reference, on the right the Scripps percent inhibition set. For each compound from NCGC the highest similarity was retained with the Scripps compounds from each category. A similar distribution was obtained with the BCI fingerprints (not shown).

We conclude that all attempts to predict NCGC from Scripps or the reverse have failed. Guha and Schürer derived bit spectra to show that the toxic class of the NCGC IC50 set is equally similar to the toxic and non-toxic class of the Scripps IC50 [11]. This was used to explain the failed prediction of Scripps results by a model generated from the NCGC data. In Figure 6 the FCFP_6 similarity distribution is shown between the toxic compounds from the NCGC set compared to the toxic and non-toxic compounds from the Scripps IC50 and percent inhibition sets. The NCGC toxic set is dissimilar to both the Scripps IC50 toxic and non-toxic compounds. When the NCGC toxic compounds are compared to the larger Scripps percent inhibition set, the similarity to the non-toxic compounds has increased slightly, partly due to the disproportionally larger number of non-toxic compounds in this set. The NCGC toxic compounds are also dissimilar to this larger Scripps set, which explains why a model derived from the latter is also not predictive for NCGC.

**Figure 7:**
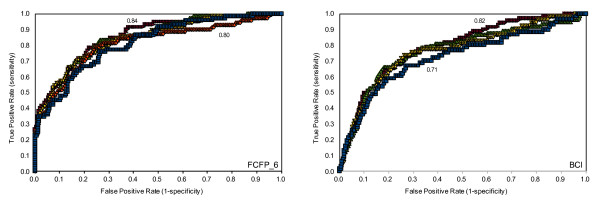
**Comparison of ROC plots for models derived using different fingerprints**. ROC plots of the 5 fold cross-validated NCGC/Scripps IC50 merged model using FCFP_6 (left) and BCI (right) fingerprints.

Since both assay formats were similar and we observed an increase in predictive power when all Scripps percent inhibition data were included, in all likelihood the NCGC and Scripps assays should be predictive for each other *if *there is sufficient overlap in chemical space. To test this hypothesis we merged the NCGC and Scripps IC50 sets into one set of 2103 compounds of which 345 are labelled cytotoxic. As with the separate NCGC and Scripps IC50 sets we built a Bayesian model with 5-fold cross validation. In Figure 7 the ROC plots are shown for the unified models built with FCFP_6 and BCI fingerprints. These models perform much better than the previous models from the individual NCGC or Scripps IC50 sets: the ROC score using the FCFP_6 fingerprints is 0.82 ± 0.02 and 0.75 ± 0.05 for the BCI fingerprints. These results clearly show that merging these two datasets has been synergistic, and therefore corroborates the hypothesis that it is only the lack of overlap in chemical space preventing better prediction scores in the separate models. However, the improvement in predictive power is unbalanced. The unified model is worse in finding the cytotoxic compounds that originated from the NCGC set (9 true positives for the unified FCFP_6 model versus 16 for the NCGC model), but better in identifying the true positives originating from the Scripps set (218 versus 161). The unified model identifies more true positives (and better ROC scores) because the number increased more in the Scripps set than it decreased in the NCGC set. Merging the sets is equivalent to adding more inactives to the Scripps set since the hit rate of NCGC is much lower at 4.7% compared to Scripps at 37%. In Bayesian statistics the probability of a compound being cytotoxic is compared to the baseline occurrence of cytotoxicity, mixing data sets with significantly different baseline hit rates will potentially yield unbalanced models as observed here.

### Modelling Pfizer Data

The results obtained modelling the Scripps and NCGC sets using naïve Bayesian were comparable to the result obtained by Guha and Schürer using Random Forest models. Since Bayesian models do not need rebalancing of training sets with toxic/non-toxic ratios far from 1/1 we decided to use Bayesian models to analyse Pfizer data. We consistently obtained better results using FCFP_6 fingerprints than with BCI fingerprints and therefore decided to subsequently only use FCFP_6 fingerprints. We concluded from modelling the Scripps data that Bayesian models can improve if *all *percent inhibition data are used to augment the data set and that a much lower cut-off can be used than is typically applied by the experimenter. The Pfizer data set contains results from 33 assays with percent inhibition data and 52 assays with IC50 data. These data have been obtained by Pfizer and its multiple legacy companies and not surprisingly a variety of assay formats have been applied. We developed assay meta data collection tools for the biological assays to focus on the factors most likely to influence cytotoxicity (*e.g. *cell-line, incubation time, dose, endpoint detection method). Extensive data profiling was applied to generate a well characterised data set (***Pfizer dataset collection and profiling - Methods***).

Many of the Pfizer assays were selectivity assays, aimed at removing "actives" from the primary assay where the activity was in fact due to cytotoxicity or another non-specific event. Since the compounds submitted to these assays had already shown activity in a cell-based assay, they are not true random subsets of the Pfizer file and the expected toxic hit rate is closer to the Scripps IC50 set (37%) than to the Scripps percent inhibition set (1.4%). The cytotoxicity assay collection also covered different % inhibition and IC50 dose ranges. A particular cut off may give 20% actives in one assay, but 100% actives in another. Therefore to enable cross-assay comparison, the top 20% of compounds (by activity or pIC50) were considered active so that every assay would have the same hit rate. For an assay with a normal distribution this would equal mean plus (just under) one standard deviation. Modelling the Scripps percent inhibition data has shown that including this many actives in the training set can still yield a predictive model. An important feature of Bayesian learning is that it is not sensitive to the ratio of actives in the dataset; the ROC scores in Figure 2 illustrate this point: essentially the same model is obtained from the Scripps percent inhibition data, whether the cut-off for activity is set to 10% or to 80% or to any value in between. This advantage of Bayesian learning means we can pragmatically define the top 20% of compounds as toxic without decreasing the quality of the model.

Our aim was to derive one generally applicable cytotoxicity model and it was therefore tempting to integrate all data into one training set, hoping for a synergy in predictive power similar to that observed when the NCGC and Scripps IC50 sets were combined. We decided to take a more systematic approach and to only include data sets leading to models that are predictive for at least one other data set.

For each assay with at least 10 toxic molecules, a Bayesian model was derived and the ROC scores were calculated predicting the outcome of each of the other assays. To visualise connections between data sets prediction networks were created. (***see Prediction Networks - Methods)***

### Prediction Networks

In a prediction network the nodes represent data from different assays, and the size of the node is proportional to the number of molecules in the corresponding data set. Nodes are considered predictive if the model yields a ROC score greater than or equal to 0.60. Two nodes are connected if the data at one node can be used to build a predictive model for the cytotoxicity of the molecules at the other node. The nodes are only connected if predictions are bi-directional.

**Figure 8:**
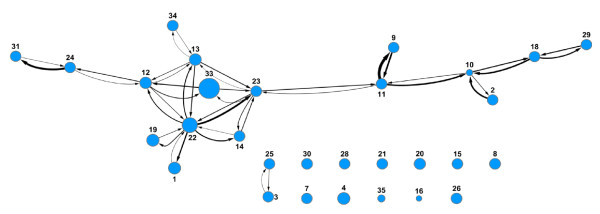
**Prediction network of percent inhibition models**. Nodes represent assays with arbitrary assay number. Node size is proportional to number of molecules in assay. The presence of edges between two nodes indicates that a model from one set is predictive for the other and vice versa. All data sets are Pfizer assays except for 33 (Scripps) and 34 (NCGC). Assays with fewer than 10 actives were removed.

**Figure 9:**
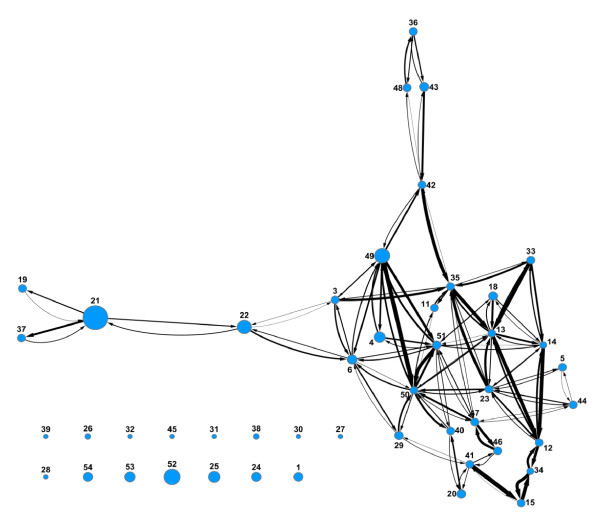
**Prediction network of IC50 models**. Nodes represent assays, with arbitrary assay number. Node size is proportional to number of molecules in assay. The presence of edges between two nodes indicates that a model from one set is predictive for the other and vice versa. All data sets are Pfizer assays except for 53 (NCGC) and 54 (Scripps). Assays with fewer than 10 actives were removed.

The percent inhibition and IC50 prediction networks are shown in Figures 8 and 9 respectively. The edges connecting the nodes have an arrow indicating the direction of the prediction from the training set to the test set. The width of the edges is proportional to the ROC score. Differences in distances between nodes have no meaning. In both networks there is one main cluster of nodes connected to each other, showing that most models derived from these assays are predictive for at least one other model. As described previously, the Scripps and NCGC IC50 data sets were not mutually predictive and their nodes are not connected to each other or indeed to any other node (nodes 53 and 54 in Figure 9). However, the Scripps and NCGC nodes are connected to other assays in the percent inhibition prediction network (nodes 33 and 34 in Figure 8). This situation also occurs with other assays in the prediction networks, the assay pair (A.B) are mutually predictive, and pair (B,C) is also mutually predictive, but (A,C) is not as with the Scripps and NCGC data sets, this could be due to a lack in overlap in chemical space between assays A and C. Although there is enough overlap between (A,B) and (B,C) for the pairs to be mutually predictive, the pair (A,C) are too far apart in chemical space to be predictive. The nodes in the IC50 network are more inter-connected than in the percent inhibition network. This is not surprising since a higher percentage of true actives can be expected in the IC50 set compared to the percent inhibition set if the first is the follow up for the latter. Even with multiple mechanisms leading to toxicity, each of which coming with a different pharmacophore, the true actives are expected to be more like each other than random compounds and cross prediction should be easier.

### One Predictive Cytotoxicity Model

Although further investigation is required to determine why some assays are predictive of each other and some are not, it is worth examining the effect of utilising the information gained from our prediction networks to derive one predictive cytotoxicity model. The 17 connected assays in the percent inhibition prediction network were combined into a training set to derive the percent inhibition cytotoxicity model. The same was done for the 31 connected screens in the IC50 prediction network to derive the IC50 cytotoxicity model. The models were derived with the same descriptors and definition of cytotoxicity as the models built when constructing the prediction networks. These models were evaluated using a 5-fold cross-validation method as before. The ROC scores from the cross validation are shown in Table 8. The models have good ROC scores and the variance in ROC score between each cross-validation is also much smaller than that observed for the earlier Scripps and NCGC models.

**Table 8:** ROC scores from the 5-fold cross-validation of the models derived from the predictive assays

Model	ROC score
Percent inhibition cytotoxicity model	0.846 ± 0.003
IC50 cytotoxicity model	0.836 ± 0.002
Merged Percent inhibition/IC50 model	0.842 ± 0.002

**Figure 10:**
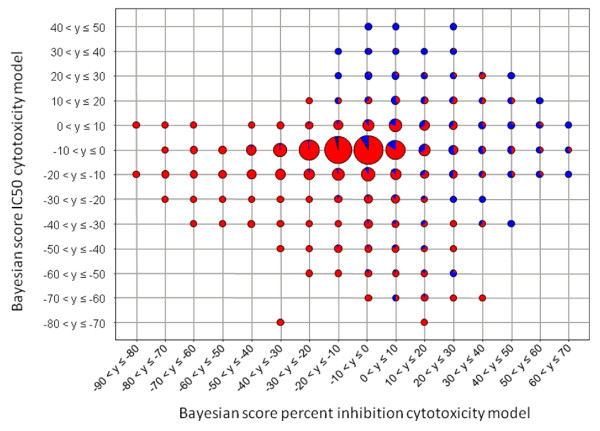
**Correlation of Bayesian scores of a test set calculated from the IC50 and percent inhibition models**. The test set consisted of 10% of the molecules from the percent inhibition cytotoxicity model training set and the IC50 cytotoxicity model training set. The Bayesian scores are binned to get 16 bins. The pies represent the number of molecules within those bins with size proportional to the number of molecules. Red segments represent the proportion of non-cytotoxic molecules and blue segments represent the proportion of cytotoxic molecules. A similar plot (not shown) was obtained using all of the data in the training set.

These results show that using a prediction network allows appropriate assay data to be selected to construct a training set to derive a predictive model. It appears that using data from a diverse set of assays and employing a prediction network to select assays for inclusion in the combined model is a powerful approach. The next step was to see if these two cytotoxicity models can be combined to give an overall predictive cytotoxicity model. A test set was created containing 10% of the molecules from the percent inhibition model training set and 10% of the molecules from the IC50 model training set. The two models were re-trained with the remaining 80% of molecules. Both the percent inhibition and IC50 cytotoxicity models were tested with the new test set. The Bayesian scores for the compounds in each assay were plotted against each other to see if there was a positive correlation between the two models. The scores were binned and for each bin a pie diagram was generated showing the percentage of cytotoxic molecules (Figure 10). The majority of cytotoxic molecules are at high Bayesian scores in both the percent inhibition and IC50 cytotoxicity models. As both models score cytotoxic compounds highly and there is a positive correlation, the two models can be combined. The training sets for the percent inhibition and IC50 cytotoxicity models are combined to give a new training set used to derive a predictive cytotoxicity model. The ROC score for the 5-fold cross-validated model is 0.842 ± 0.002, between the ROC scores of the IC50 and the percent inhibition models. This is a good model with little variance in performance between the 5 test sets. Although merging the two models does not produce a model better than the two separate models, its enrichment is still high and it creates a neater tool for predicting cytotoxicity, rather than having to use two models. The merged model also covers a larger area of chemical space making it more general than the individual models.

**Figure 11:**
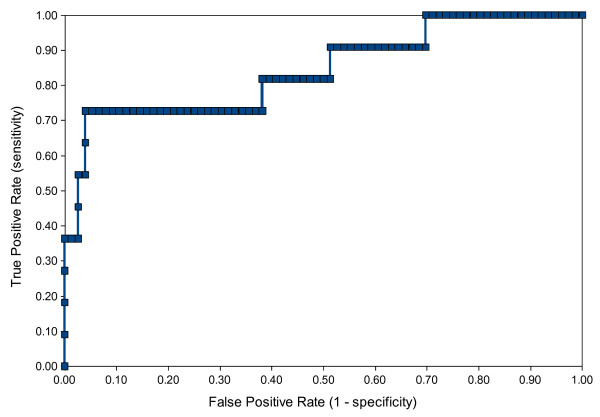
**Validating the predictive cytotoxicity model with a set of 87 FDA-approved drug compounds**. ROC plot generated from a range of FDA approved drugs

The merged model was also validated with a set of 87 drugs approved by the FDA since 2000. Approved drugs for obvious reasons are assumed to be non-cytotoxic; however we assumed the 11 drugs with an anti-cancer indication to be cytotoxic. Figure 11 shows the ROC plot for the predictive cytotoxicity model when validated with this set of drug compounds. The ROC score is 0.84, which means the model performed well at distinguishing cytotoxic drugs from other drugs.

**Figure 12:**
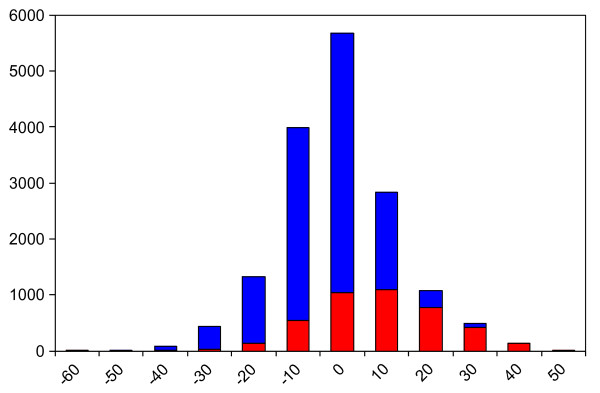
**Distribution of cytotoxicity scores generated from Pfizer model (combined IC50 and Pct inhibition)**. A range of compounds tested in an in vitro toxicity/safety assay. Actives (red), having IC50 < 50 μM, inactives (blue) with IC50 > 50 μM.

In addition, to investigate translation of cytotoxicity score to toxic effects, ~11,000 compounds which had been tested in a Pfizer *in vitro *toxicity/safety assay were tested *in silico *through the cytotoxicity prediction model. Examining the *in vitro *toxicity/safety data, at high Bayesian scores **(**Figure 12**) **there are proportionally more toxic compounds (with IC50 < 50 uM), than at the lower Bayesian scores - i.e. the activity distribution of toxic compounds with IC50 < 50 uM, centres to the right of the inactives distribution (IC50 > 50 uM), which has a lower average Bayesian score. There is therefore a good indication that compounds flagged as active in the *in vitro *toxicity/safety assay would have been identified as cytotoxic by the model.

Cytotoxicity can also be related to the descriptors used to derive the model. After the FCFP_6 fingerprints, the descriptor which has the largest impact on the Bayesian score is AlogP. Compounds having AlogP between 3.7 and 34 are given a high probability of being toxic by the model, the probability of being toxic increases at the higher end of the range. Compounds with AlogP below 3.3 are given a low probability of being toxic, generally the lower the AlogP the lower the probability of being toxic. There is one exception where an AlogP in the range of 34 to 63 gives a non toxic compound, but there is only one example of such a compound occurring, therefore this is an anomalous result. These are also unusually high values for logP; therefore AlogP is an unreliable estimate of logP for these compounds. Compounds with a high logP are lipophilic and can therefore easily cross cell membranes, their tendency to preferentially bind with proteins rather than remain in a polar solvent making them more likely to have non-specific intracellular effects. Molecular weight is the next most important descriptor. The model gives a low probability of a molecule being toxic if its molecular weight is below 370. Higher molecular weights give a high probability of toxicity. A molecule's lipophilicity will increase as its mass increases; therefore it is not surprising that heavier compounds have a higher probability of being cytotoxic. The polar surface area and the number of hydrogen bond donors and acceptors also show how cytotoxicity is dependent on the lipophilicity of the compounds.

The number of rotatable bonds also has a positive correlation with cytotoxicity score. This is to be expected, since a flexible molecule can adopt a greater number of conformations, allowing it to bind to many different sites, possibly leading to unwanted effects. Typically molecules with a large number of rotatable bonds also have a higher molecular weight - which is again correlated with logP.

The observed correlation of lipophilicity and related properties with cytotoxicity is not surprising as this has also been observed in studied linking *in vivo *toxicity[28] and bioavailability to physiochemical properties[29].

## Conclusions

There is a wealth of data from cytotoxicity assays available both publicly and within pharmaceutical companies that can be used to derive predictive models. Here, a predictive Bayesian model has been derived from public and in-house Pfizer data.

During the development of this model the need for multiple-fold cross-validations has been reinforced, as this gives the most accurate validation results. A method for cut-off optimisation has also been shown to provide an appropriate definition of cytotoxicity to build a successfully predictive model. Prediction networks have been used to make informed decisions on which data sets should be included in the training set and have identified the need for more detailed examinations of what makes two data sets predictive of each other. The prediction networks identified assay data that could be used to derive predictive models. These assays were combined into one training set that produced a successful predictive cytotoxicity model with a ROC score of 0.842 ± 0.002.

The data indicate that some assays are highly predictive of each other. We speculated that this may because they shared common assay conditions (cell line, species, incubation time, detection method etc.). To investigate this further, more networks were created in Cytoscape to incorporate the assay conditions available. However no clear relationship between these factors and cytotoxicity could be demonstrated. This does not necessarily rule out a relationship as there was little overlap in assay conditions between data sets, and only a few compounds have been tested in more than one assay. To study this hypothesis further, the prediction network method should be repeated with a dense matrix of assays spanning diverse experimental conditions and compounds tested against all assays. This information can be represented in the network and any *assay *relationships between predictive data sets will become apparent.

Although there are gaps in the understanding of why the combination of assay data used to derive the predictive cytotoxicity model works, the model is still an extremely useful tool and also supports previous evidence in the literature that toxicity is related to lipophilicity. This model could be used to triage hits from primary cell-based screens for cytotoxicity, rather than running parallel cytotoxicity assays. The model predictions track well with the *in vitro *safety/toxicology assay we examined, but the applicability of the model as a tool to help identify toxic molecules early on in the drug discovery pipeline would be increased if its output could be compared with more *in vitro *assays of this type. Once more is understood on what makes a data set predictive, this knowledge can be utilised to derive a more accurate predictive model. Modelling methods described in this paper are not limited to cytotoxicity; they can also be used when predicting other molecular properties, or compound activities.

## Experimental Methods

### Data sets

Cytotoxicity assay data from publicly available sources and Pfizer in-house screening data were used to train the Bayesian models. Four data sets were used covering 172,506 compounds from 89 assays and contain a mixture of percent inhibition and IC50 data (Table 9).

**Table 9:** Summary of the 4 data sets used to build Bayesian models to predict cytotoxicity

Data set	Source	Description	No. of assays	No. of compounds	IC50	Percent inhibition
Scripps	PubChem, AID 364, 463, 464	T-Cell (Jurkat) proliferation data containing a mixture of percent inhibition and IC50 measurements	3	60503	768	59735

NCGC	PubChem, AID 426	T-Cell (Jurkat) proliferation data containing IC50 measurements	1	1277	1277	0

Pfizer percent inhibition	Pfizer	Percent inhibition data from a variety of different cytotoxicity assays	33	83284	0	83284

Pfizer IC50	Pfizer	IC50 data from a variety of different cytotoxicity assays	52	28492	28492	0

#### Pfizer dataset collection and profiling

We conducted a gap analysis on the original dataset to identify those protocols where a substantial proportion of the assay experimental conditions was missing or inconsistent, which was the case for some legacy protocols. Examination of the full assay documents and direct contact with the biologists involved allowed us to generate a list of 82 assays with comprehensive coverage of the assay experimental parameters.

Assay endpoint detection methods were classified as Fluorescence emission, Luminescence, RNA quantification and Absorbance. The assay technologies included dye binding, flow cytometry, formazan dye formation, luciferase, PCR, and Resorufin dye formation. Data was used from a variety of species - Human, Hamster, Mouse, Pig, Rat, and Monkey - and a total of 34 different cell lines across all of the assays. To standardize the data and improve confidence in the model, the cell lines were re-classified according to their tissue origin (blood, skin, colon, cervix, ovary, lung, kidney, breast, foreskin, liver, aorta, brain, connective tissue, muscle, and nerve). Incubation times were standardised to a base unit of hours - our observations indicate that a wide range of incubation periods are used in cytotoxicity screens (2 hours to 145 hours) and they can vary within the same tissue type, or assay technology.

In addition to assay profiling and classification we analysed the percent inhibition and IC50 results for each assay to determine whether these results could be included in our models. Wherever we could not identify the convention used to distinguish cytotoxic compounds we decided to remove this data from further analysis. The assays where we could not reliably differentiate between true actives, artefacts and different naming conventions were likewise excluded.

To allow the model to make appropriate comparisons, data from the remaining HTS assays was examined to ensure there was the expected normal distribution around zero % inhibition. Assays were excluded from further analysis where this was not the case. Assay results where the endpoint value violated the standard business rules (*e.g*. zero or null) were also excluded. Scitegic Pipeline Pilot was used to develop an automated data cleaning tools to perform the tasks described in this section. In addition, using curve fit descriptors and quality parameters, we generated Spotfire plots and screen data confidence scores [30] which enable interactive exploration and assessment of the data quality. These tools were used to refine the IC50 data set to a list of 52 assays where the data, curve fits and endpoints were reliable and well understood.

#### Bayesian Learning and Bayesian score

Pipeline Pilot[31,32] was used to perform all calculations. During the period of research versions 6.5, 7.0 and 7.5 were used, but there are no differences in the components used in these versions. Bayesian learning is based on Bayes' rule for conditional probability which gives the probability of an event A occurring given that event B has already occurred. In a cytotoxicity context, this is the probability of a compound being toxic, given that it contains a particular descriptor. For each descriptor, D, the probability of a molecule being toxic given it contains descriptor D is calculated as P(Active|;D) = A_D_/(A_D_+I_D_), where A_D _is the number of active compounds containing descriptor D and I_D _is the number of inactive compounds containing descriptor D. These probabilities become unreliable as the number of molecules containing descriptor D becomes small. Therefore a Laplacian modified model is derived which takes into account the different sampling frequencies of different features by adding samples with the same hit rate as observed in the training set.

#### Laplacian modified model

If we assume most features have no relationship to activity then we would expect P(active|;D) to be equal to the overall activity rate, P(active) = A/(A+I). If we sample a feature K additional times, where K = 1/P(active), we would expect P(active)K of these samples to be active. Therefore the Laplace corrected probability of a compound being active given a certain descriptor D, P(Active|;D), is equal to (A_D_+P(active)*K)/((A_D_+I_D_)+K). As (A_D_+I_D_) approaches 0 the feature probability converges towards P(active) which is expected if it is assumed the feature has no relationship to activity. The Bayesian score calculated for a compound of unknown class is calculated by multiplying the probabilities for each descriptor contained in the compound; this score represents the likeliness of the compound being active.

### Model Building

#### Percent inhibition cut-off optimisation

The following method was used to find the best percent inhibition value to use as the definition for cytotoxicity for the molecules in the Scripps data set. The best cut-off is the value that gives the highest ROC score when used to build a model. The ROC score is the area under the curve of the ROC plot for the model. This method was originally suggested by David Rogers [27]. A set of 121 models was built, each with a different percent inhibition cut-off as the definition for toxicity. The cut-offs ranged from -20% to 100% in 1% increments. The ROC score was calculated for each of these models and was plotted against the corresponding cut-off. The optimum cut-off is defined as the cut-off that yields the highest ROC score. As 5-fold cross-validation is used to test the models, the same method is also used in the cut-off optimisation. The set of 121 models is trained on 80% of the data and the ROC scores are calculated by testing on the remaining 20% of the data. This is repeated 5 times using a different 20% to test the model each time. When the ROC score is calculated the cytotoxic compounds are defined as those that were labelled as active in the original data extracted from PubChem. These labels were assigned based on the percent inhibition or IC50 values if available for the molecules. This procedure was repeated twice. Once for the FCFP_6 fingerprints and once for the BCI fingerprints.

#### Prediction networks

A major challenge for machine learning methods is to understand the applicability domain of models. For example a model trained on a particular data set may perform well when cross-validated, but fail at classifying compounds from a different data set. This research aims to determine which assay data can be used to predict the outcome of other assays and to understand any relationship between such data sets. To do this we have created prediction networks.

The available Pfizer data were split into two categories: IC50 and percent inhibition data. This is because IC50 data are often obtained as confirmations of previous data and are therefore enriched in hit rate but with lower chemical diversity of compounds (as was the case with the Scripps data). The hit rate for the Pfizer assays was artificially set to 20%, but the chemical diversity has probably been artificially lowered by routinely removing compounds with undesirable chemical functional groups and/or physical properties. The NCGC and Scripps data were included as well as separate screens. There are no distinct percent inhibition measurements available for NCGC, therefore we took the percent inhibition at 9.2 μM from the full curve data as a surrogate.

The Pfizer percent inhibition data set contains data from 33 assays, A Bayesian model was derived for each assay, giving a total of 28 models (5 of the assays contained only 1 molecule so a model could not be trained). Each of these models was then tested in turn with data from the remaining assays not used to train the model. Each of the models was also tested on the Scripps percent inhibition and NCGC percent inhibition data sets, and the Scripps percent inhibition and NCGC models were be tested with each of the Pfizer percent inhibition models. A text delimited file was created containing a column for training set, a column for test set and a column for the ROC score when a model trained with the training set, is tested with the test set. This file was imported into Cytoscape v.2.6.1[33] where the prediction networks were created.

The same method was applied to the 52 assays in the Pfizer IC50 data set. A total of 45 models were produced as 7 of the assays only contained 1 molecule. The Scripps IC50 and NCGC data sets were also included. For all models built, FCFP_6 fingerprints, AlogP, number of hydrogen bond donors, number of hydrogen bond acceptors, number of rotational bonds and molecular fractional polar surface area were used as descriptors. Since for most of the assays it had not been recorded what constitutes as a cytotoxic outcome the top 20% compounds (top percent inhibition or top pIC50) of each assay were classed as toxic. For the Scripps and NCGC data sets the definitions for toxicity described above were used.

Two prediction networks were built, one for the Pfizer percent inhibition data set and one for the Pfizer IC50 data set. Assays are represented in the network as nodes, and the nodes are connected with an edge if a model trained with the screen at the source node is successful in predicting the cytotoxicity of the screen at the target node as defined by a ROC score greater than 0.60. The networks are arranged using a spring-embedded layout. A spring-embedded layout positions nodes to give an aesthetically appealing layout. This is done by replacing the nodes with rings and each edge with a spring. The nodes are placed in an initial layout then are let go so the springs force the nodes to move to a minimal energy layout.

## Competing interests

The authors declare that they have no competing interests.

## Authors' contributions

SRL performed most calculations and wrote the first draft of this manuscript. JM suggested examining the Pfizer internal cytotoxicity data to develop a predictive model. GVP and JM collected, profiled and cleaned the Pfizer internal data. WPvH designed the study and performed some of the calculations. All authors contributed to the final version of the manuscript.
